# Editorial: Role of Molecular Modulators in Combatting Cardiac Injury and Disease: Prevention, Repair and Regeneration

**DOI:** 10.3389/fcvm.2022.861442

**Published:** 2022-04-18

**Authors:** Lisandra E. de Castro Brás, Ryan S. Schibalski, Daria V. Ilatovskaya, Caitlin C. O'Meara, Kristine Y. DeLeon-Pennell

**Affiliations:** ^1^Department of Physiology, The Brody School of Medicine, East Carolina University, Greenville, NC, United States; ^2^Department of Physiology, Augusta University, Augusta, GA, United States; ^3^Department of Physiology, Cardiovascular Center, Genomics Sciences and Precision Medicine Center, Medical College of Wisconsin, Milwaukee, WI, United States; ^4^Department of Medicine, Division of Cardiology, Medical University of South Carolina, Charleston, SC, United States; ^5^Ralph H. Johnson Veterans Affairs Medical Center, Charleston, SC, United States

**Keywords:** cardiovascular disease, regeneration, remodeling, inflammation, extracellular matrix

Cardiovascular disease (CVD) is the leading cause of death in the United States with heart failure (HF) being the highest reason for hospital admission. Despite improved therapies for CVD patients, the 5-year mortality rate after HF hospitalization remains around 40% (1). Preclinical and clinical studies have attempted to promote healing and decrease HF incidence in high-risk patients. While great strides have been made, significant knowledge gaps in our understanding of cardiac repair and regeneration remain. Advanced interpretation of the molecular mechanisms that stimulate beneficial vs. adverse remodeling is critical for improving current therapies.

In the current digest topic (https://www.frontiersin.org/research-topics/18185/role-of-molecular-modulators-in-combatting-cardiac-injury-and-disease-prevention-repair-and-regenera#overview), authors identify possible mechanisms for prevention, repair, and cardiac regeneration. Here, we summarize the major findings of interest to the readership and provide a frame of reference for future studies.

Over 50% of HF patients present with preserved ejection fraction (HFpEF), a prevalent pathology with no specific therapy (2). Recent molecular and cellular studies provide evidence that HFpEF is not a homogenous disease, instead, it presents through heterogeneous pathophysiology with aging as a common denominator. Superimposed with aging, obesity activates multiple inflammatory pathways that intersect with metabolic dysfunction and exacerbates uncontrolled, non-resolving, inflammation in HFpEF patients. The review by Tourki and Halade compiles current literature on obesity-driven HFpEF and discusses the potential of formyl peptide 2 receptor, an essential molecule for resolution of inflammation post-cardiac injury, as a prospective target to promote tissue clearance and expedite cardiac repair and regeneration. The authors stress the importance and benefit of an appropriate diet and nutrient intake as a preventative tool for development and progression of HFpEF.

Inflammation plays a central role in CVD. However, therapeutics that target inflammatory mediators have not been effective, likely because a controlled inflammatory response is necessary for repair and regeneration (3, 4). Rech and Rainer describe emerging evidence of the therapeutic potential for the innate immune DNA sensor cyclic GMP-AMP synthase (cGAS) and stimulator of interferon genes (STING) pathway in CVD. Many of the risk factors associated with CVD including smoking, obesity, and aging are accompanied by alterations in cGAS-STING signaling (5–9). Inhibition of cGAS and STING activation has been shown to be beneficial in CVD ranging from MI to models of HFpEF (10–12). While the data is promising, Rech and Rainer indicate concern that long-term inhibition of the cGAS-STING pathway could promote cancer or viral infection.

Nicotinamide adenine dinucleotide (NAD) is an essential cellular substrate critical for energy production. A decrease in NAD^+^ abundance has been associated with metabolic stress, chronic inflammation, and aging (13, 14). The review by Jahan and Bagchi emphasizes NAD^+^ as a promising therapy for reducing CVD risk through its actions on inflammation, muscle function, and mitochondrial health. Highlighting clinical trials such as NCT02921659 (15), the authors underline that direct and indirect NAD^+^ supplementation (by either increasing NAD^+^ precursors, e.g., tryptophan or nicotinic acid, or inhibiting NAD^+^ processing enzymes) is associated with beneficial outcomes such as decreased blood pressure and aortic stiffness, improved hypercholesterolemia, and enhanced cardiac mitochondrial function (15–18). Jahan and Bagchi stress that although boosting NAD^+^ levels is promising both for therapy and prevention of CVD, the type of NAD^+^ supplementation, as well as the dosage, should be critically evaluated to ensure both the effectiveness of treatment and prevention of potential side effects.

The ECM from neonatal hearts has pro-regenerative properties compared to that of the adult heart (19–22). Dissecting the bioactive vs. biomechanical aspects of the ECM has been challenging and has limited our understanding of ECM effects on cardiac regeneration. Wang et al. thoroughly explored the role of heart stiffness, ECM proteins, and the combination of these factors on the cardiac regenerative response in juvenile mice. The investigators administered β-aminopropionitrile (BAPN) or genipin to alter tissue stiffness before subjecting mice to myocardial infarction (MI) at postnatal day 5. After MI, mice were given decellularized ECM (dECM) derived from either fetal or adult pigs. Consistent with published literature, fetal heart dECM produced pro-regenerative phenotypes including improved ejection fraction, reduced scarring, and increased cardiomyocyte cell cycle activity post-MI. Of particular novelty, the effects of fetal dECM were substantially accentuated when tissue stiffness was decreased by BAPN administration, suggesting an interaction between bioactivity and biomechanics in cardiac repair. Future studies identifying the specific ECM factors mediating biomechanical transduction pathways and cardiac regeneration will pave the way for new therapeutic approaches in post-MI patients.

The principal functions of the heart are regulated by the autonomic nervous system of which dopamine acts as an important neurotransmitter by stimulating peripheral dopamine receptors including D1R and D3R (23, 24). Kisling et al. report for the first time the existence of an intrinsic cardiac dopaminergic system as demonstrated by both D1R and D3R expression in murine cardiac tissue and fibroblasts. Mice with dysfunctional D3R displayed limited fibroblast proliferation and migration, reduced viability, and increased expression of collagen type 3. These phenotypes were recapitulated using a non-ergot pharmacological inhibitor of D3R. While a large body of evidence describes roles for dopamine and its receptors in the neuro and renal-vascular systems (25–31), there is very little information on the functions of these receptors in the heart. Data described in this brief report points to a potential role for the dopaminergic system in cell apoptosis and cardiac fibrosis, making this system of interest when studying modulation of cardiac repair and remodeling.

This editorial commentary highlights the key points from the collection of review and original research articles in the current topic issue (Figure 1). The phenotype of CVD is broad and diverse; thus, defining each pathophysiological process and understanding what factors contribute to repair and regeneration is needed for improvement in prognosis. The research community should strive to identify the correct balance of molecular triggers that limit adverse remodeling and HF pathogenesis by inhibiting an exacerbation of inflammation and ECM accumulation and promoting reparative processes. In addition, consideration for the role that primary risk factors such as gender, aging, obesity, and drug interactions have on the multiple molecular regulators of cardiovascular remodeling is warranted.

**Figure 1:**
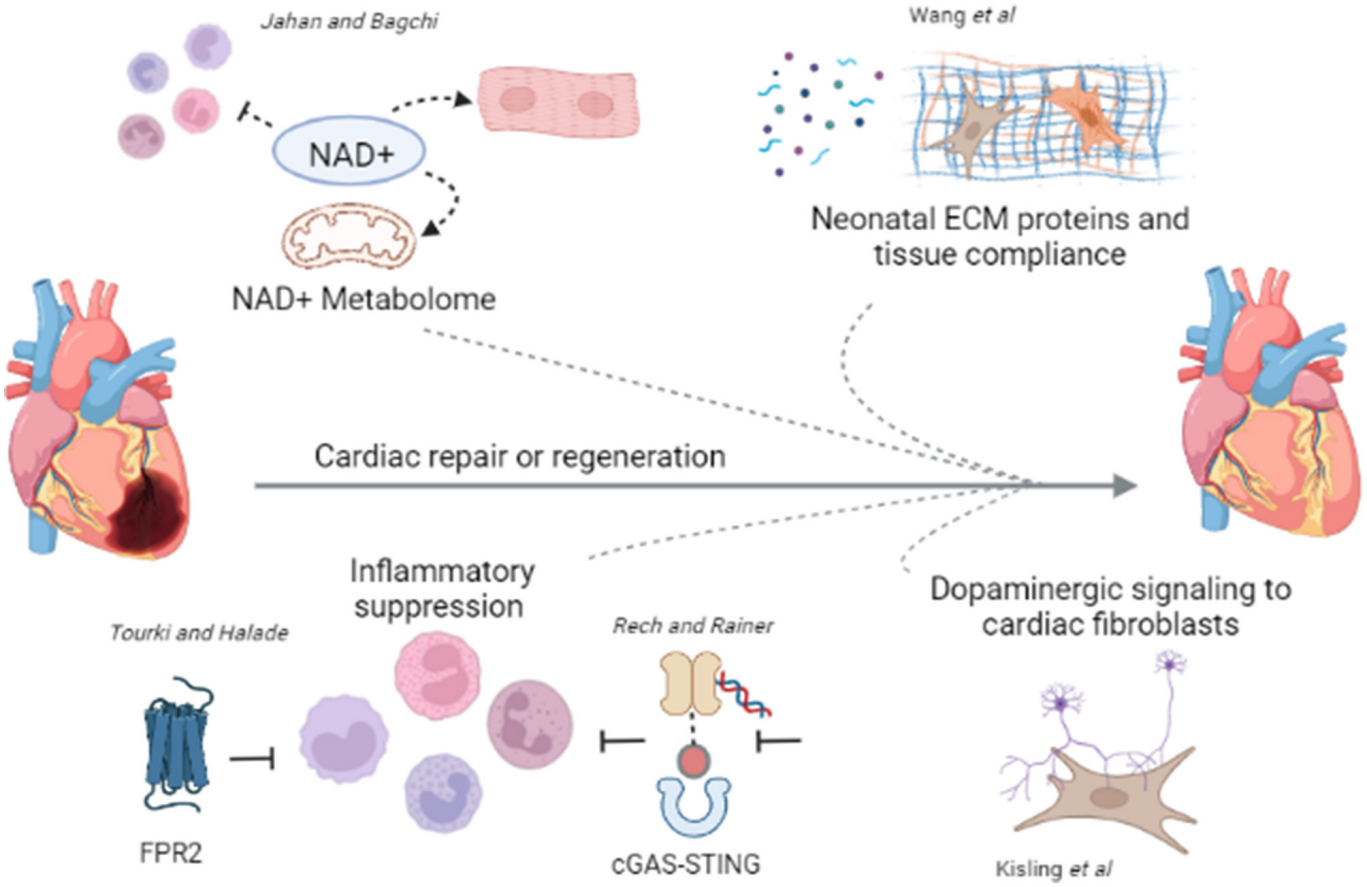
Broad and diverse mechanisms contribute to cardiac repair or regeneration. In the current topic issue, articles review the literature regarding NAD^+^ metabolome in CVD (Jahan and Bagchi), and inflammatory suppression *via* FRP2 activation (Tourki and Halade) or cGAS-STING inhibition (Rech and Rainer) in cardiac repair. Primary research articles demonstrate a role for ECM proteins and tissue compliance in cardiac regeneration (Wang et al.), and dopaminergic signaling to cardiac fibroblasts in cardiac repair post MI (Kisling et al.). Future studies should assess what molecular triggers can tip the balance to limit adverse remodeling and the pathogenesis of HF promoting the reparative processes. Figure was generated using Biorender.com.

## Author Contributions

CO'M prepared the figure. All authors conceived, drafted, edited the manuscript, and approved this final manuscript.

## Funding

We acknowledge funding from the National Institutes of Health under Award Numbers HL148114 (DI), HL145817 (KD-P), HL156022 (CO'M), HL141159 (CO'M), and HL152297 (LdCB), the Biomedical Laboratory Research and Development Service of the Veterans Affairs Office of Research and Development under Award Number BX003922 (KD-P), Advancing a Healthier Wisconsin Endowment (AHW) #5520561 (CO'M), American Heart Association IPA35260039 (KD-P), and the Department of Physiology startup funds from Augusta University (DI).

## Author Disclaimer

The content is solely the responsibility of the authors and does not necessarily represent the official views of any of the funding agencies.

## Conflict of Interest

The authors declare that the research was conducted in the absence of any commercial or financial relationships that could be construed as a potential conflict of interest.

## Publisher's Note

All claims expressed in this article are solely those of the authors and do not necessarily represent those of their affiliated organizations, or those of the publisher, the editors and the reviewers. Any product that may be evaluated in this article, or claim that may be made by its manufacturer, is not guaranteed or endorsed by the publisher.
